# Microvascular Dysfunction in Patients With Type II Diabetes Mellitus: Invasive Assessment of Absolute Coronary Blood Flow and Microvascular Resistance Reserve

**DOI:** 10.3389/fcvm.2021.765071

**Published:** 2021-10-19

**Authors:** Emanuele Gallinoro, Pasquale Paolisso, Alessandro Candreva, Konstantinos Bermpeis, Davide Fabbricatore, Giuseppe Esposito, Dario Bertolone, Estefania Fernandez Peregrina, Daniel Munhoz, Niya Mileva, Martin Penicka, Jozef Bartunek, Marc Vanderheyden, Eric Wyffels, Jeroen Sonck, Carlos Collet, Bernard De Bruyne, Emanuele Barbato

**Affiliations:** ^1^Cardiovascular Center Aalst, OLV-Clinic, Aalst, Belgium; ^2^Department of Translational Medical Sciences, University of Campania ‘Luigi Vanvitelli', Naples, Italy; ^3^Department of Advanced Biomedical Sciences, University of Naples Federico II, Naples, Italy; ^4^Discipline of Cardiology, Department of Internal Clinical Medicine, University of Campinas, Campinas, Brazil; ^5^Department of Cardiology, Lausanne University Hospital, Lausanne, Switzerland

**Keywords:** diabetes mellitus, coronary microvascular dysfunction (CMD), coronary flow reserve (CFR), microcirculatory resistance, continuous thermodilution technique

## Abstract

**Background:** Coronary microvascular dysfunction (CMD) is an early feature of diabetic cardiomyopathy, which usually precedes the onset of diastolic and systolic dysfunction. Continuous intracoronary thermodilution allows an accurate and reproducible assessment of absolute coronary blood flow and microvascular resistance thus allowing the evaluation of coronary flow reserve (CFR) and Microvascular Resistance Reserve (MRR), a novel index specific for microvascular function, which is independent from the myocardial mass. In the present study we compared absolute coronary flow and resistance, CFR and MRR assessed by continuous intracoronary thermodilution in diabetic vs. non-diabetic patients. Left atrial reservoir strain (LASr), an early marker of diastolic dysfunction was compared between the two groups.

**Methods:** In this observational retrospective study, 108 patients with suspected angina and non-obstructive coronary artery disease (NOCAD) consecutively undergoing elective coronary angiography (CAG) from September 2018 to June 2021 were enrolled. The invasive functional assessment of microvascular function was performed in the left anterior descending artery (LAD) with intracoronary continuous thermodilution. Patients were classified according to the presence of DM. Absolute resting and hyperemic coronary blood flow (in mL/min) and resistance (in WU) were compared between the two cohorts. FFR was measured to assess coronary epicardial lesions, while CFR and MRR were calculated to assess microvascular function. LAS, assessed by speckle tracking echocardiography, was used to detect early myocardial structural changes potentially associated with microvascular dysfunction.

**Results:** The median FFR value was 0.83 [0.79–0.87] without any significant difference between the two groups. Absolute resting and hyperemic flow in the left anterior descending coronary were similar between diabetic and non-diabetic patients. Similarly, resting and hyperemic resistances did not change significantly between the two groups. In the DM cohort the CFR and MRR were significantly lower compared to the control group (CFR = 2.38 ± 0.61 and 2.88 ± 0.82; MRR = 2.79 ± 0.87 and 3.48 ± 1.02 for diabetic and non-diabetic patients respectively, [p < 0.05 for both]). Likewise, diabetic patients had a significantly lower reservoir, contractile and conductive LAS (all *p* < 0.05).

**Conclusions:** Compared with non-diabetic patients, CFR and MRR were lower in patients with DM and non-obstructive epicardial coronary arteries, while both resting and hyperemic coronary flow and resistance were similar. LASr was lower in diabetic patients, confirming the presence of a subclinical diastolic dysfunction associated to the microcirculatory impairment. Continuous intracoronary thermodilution-derived indexes provide a reliable and operator-independent assessment of coronary macro- and microvasculature and might potentially facilitate widespread clinical adoption of invasive physiologic assessment of suspected microvascular disease.

## Introduction

Type 2 diabetes mellitus (DM) is a common comorbidity in the general population with prevalence increasing worldwide, frequently diagnosed in patients with cardiovascular diseases (1, 2). Despite the improvement of medical treatment and lifestyle changes, diabetic patients still present an increased risk for microvascular and macrovascular complications compared to non-diabetic subjects (3). A large contribution to morbidity and mortality in diabetic patients can be attributed to the accelerated development of obstructive coronary artery disease (CAD) (4). Moreover, it is widely acknowledged that coronary microvascular dysfunction (CMD) is an early feature of DM that may precede macrovascular disease and constitutes a key feature of diabetic cardiomyopathy (5–8). Nevertheless, the presence and related pathological mechanisms underlying microvascular dysfunction in diabetic patients are still under-reported also following the limited ability to reliably assess the microcirculation in patients.

Coronary flow reserve (CFR) is a surrogate marker of microvascular function in patients without significant epicardial coronary artery stenosis and has been used to disclose impaired microcirculation in diabetic patients by the means of Doppler flow velocities or bolus thermodilution (9–11); yet these latter techniques are associated with large variability both patient and operator-dependent that have limited their clinical widespread adoption (12, 13). Recently, technical advances allowed to obtain an accurate (14) and reproducible (15) evaluation of absolute coronary flow and microvascular resistance by continuous intracoronary thermodilution through a dedicated microcatheter, thus introducing an alternative to Doppler or bolus thermodilution measurements (16). Microvascular Resistance Reserve (MRR), derived from continuous intracoronary thermodilution, is a novel index specific for microvasculature, independent of autoregulation and myocardial mass, based on absolute values of coronary flow and pressures (17).

The present study sought to compare absolute coronary flow, microvascular resistance and MRR, assessed by continuous intracoronary thermodilution in both diabetic and non-diabetic patients.

## Methods

### Patients Included

In this observational retrospective study, consecutive patients undergoing elective coronary angiography (CAG) from September 2018 to June 2021 with suspected angina and non-obstructive coronary artery disease (NOCAD) and subsequent invasive functional assessment of microvascular function with intracoronary continuous thermodilution were considered eligible.

Inclusion criteria were: (1) assessment of both resting and hyperemic absolute flow and resistance in the left anterior descending artery (LAD); (2) the absence of significant epicardial stenosis in the vessel investigated (defined as diameter stenosis [DS] > 50% by visual estimation).

Exclusion criteria were the presence of a previous myocardial infarction (MI) in the LAD territory, a functional assessment made immediately after PCI, acute decompensated heart failure, acute coronary syndrome, severe valvular disease, and hypertrophic cardiomyopathy.

Pre-existing type II diabetes mellitus (DM) was defined as a known reported history of DM at admission, either treated with diet and lifestyle measures alone or with the additional use of oral glucose-lowering medications and insulin (18). Diagnosis of DM was achieved during hospitalization based on fasting plasma glucose levels ≥ 126 mg/dL, or glycosylated hemoglobin (HbA1c) ≥ 48 mmol/mol (therapeutic target of T2DM was HbA1c >53 mmol/mol). Patients without a history of DM and with HbA1c <48 mmol/mol were considered non-diabetic.

The protocol was approved by the institutional review board, and the study was conducted in accordance with regulatory standards. All patients provided their written informed consent.

### Procedure

Coronary angiography was performed through either radial or femoral access. A 6Fr guiding catheter was used and 0.2 mg of Isosorbide Dinitrate was administered intracoronary. A guidewire equipped with a pressure/temperature sensor (PressureWire X, Abbott, IL) was connected to a dedicated software for tracings analysis (Coroventis CoroFlow Cardiovascular System, Uppsala, Sweden) and, after zeroing, was advanced through the guiding catheter. The pressures recorded by the pressure/temperature wire and by the fluid-filled guide catheter were equalized close to the tip of the guiding catheter. Next, the wire was advanced into the distal part of the coronary artery and the temperature zeroed. Subsequently, a dedicated monorail infusion 2.52 F catheter with 4 lateral side holes (RayFlowTM, Hexacath, Paris, France) was advanced over the pressure/temperature wire and connected to the 200 cc syringe of an automated injection system (Medrad® Stellant, Medrad Inc, Warrendale, PA, USA) filled with saline at room temperature (typically between 20 and 23°C). The infusion catheter was advanced into the artery to be investigated and its tip positioned into the first millimeters of the vessel.

### Microcirculation Assessment With Intracoronary Continuous Thermodilution

Microvascular function was assessed with continuous intracoronary thermodilution of saline at room temperature. Absolute coronary flow (Q, mL/min) as derived from continuous thermodilution was calculated by the previously validated equation (19):


$$\begin{array}{l} Q=1.08\;\cdot \;\frac{T_{i}}{T}\;\cdot Q_{i} \end{array}$$

**Figure 1:**
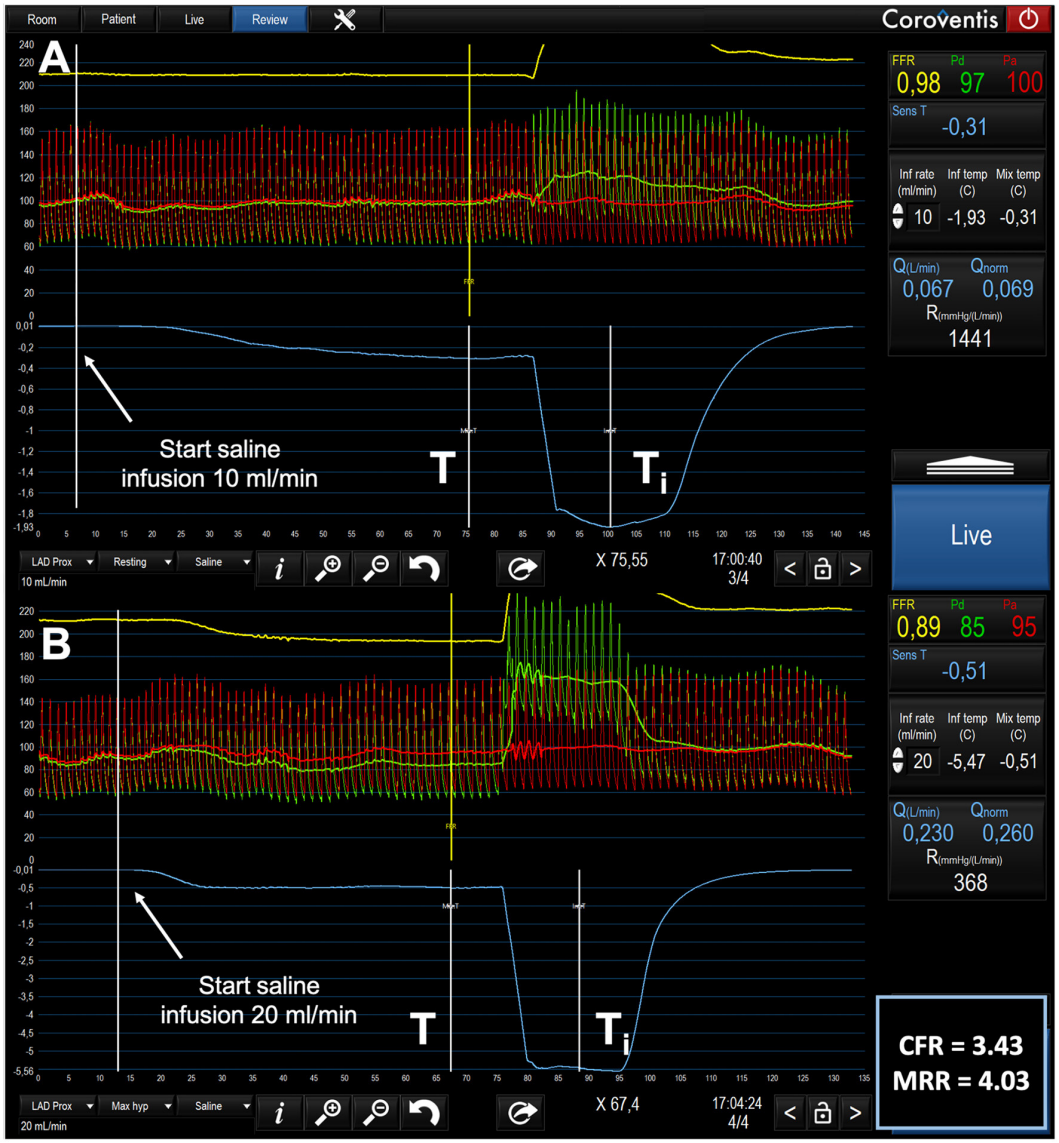
Example of tracings of phasic and mean central aortic pressure (Pa), distal coronary pressure (Pd) and thermodilution during the infusion of 10 mL/min **(A)** and of 20 mL/min **(B)** in the LAD of a 77-year-old male patients with mild wall irregularities. **(A)** after the start and during the next 70 s of the infusion of saline at 10 mL/min through the RayFlow^TM^ catheter located in the proximal LAD, no changes in Pd / Pa were observed. Resting flow is 67 mL/min. **(B)** in contrast, after the start and during the next 70 s, the infusion of saline at 20 mL/min through the RayFlow^TM^ catheter located in the proximal LAD, was paralleled by a decrease in Pd and in Pd/Pa. Hyperemic flow is 230 mL/min. CFR and MRR are 3.43 and 4.03 respectively. Q, Absolute Flow; Qnorm, Normalized Absolute Flow (Q/FFR); R, Absolute Microvascular Resistance; CFR, Coronary Flow Reserve; MRR, Microvascular resistance Reserve.

In which Q_*i*_ is the infusion rate of saline by the infusion pump (in mL/min); T_*i*_ is the temperature of the infused saline when it exits the infusion catheter and T is the temperature of the mixture of blood and saline in the distal part of the coronary artery during steady state infusion. T_*i*_ and T are both expressed relative to the patient's blood temperature before the start of the infusion. The constant 1.08 is related to the difference between the specific heats and densities of blood and saline when saline is infused in blood. An example of thermodilution tracings during the infusion of 10 mL/min and of 20 mL/min is given in the Figure 1.

Resting absolute coronary (Q_rest_) flow was measured with saline infusion at 10 mL/min, whilst hyperemic flow (Q_hyp_) was measured with saline infusion at 20 mL/min (20). Absolute resistance at rest (R_μ−*rest*_) and during hyperemia (R_μ−*hyp*_)—in Woods Units (WU)—was calculated as the ratio between the distal coronary pressure during each infusion (P_d_) and Q_rest_ or Q_hyp_, respectively.

Fractional Flow Reserve (FFR) was calculated as the ratio between distal coronary pressure and central aortic pressure (P_d_ and P_a_, respectively) during saline-induced hyperemia.

Coronary Flow Reserve (CFR) was defined as the ratio between absolute hyperemic flow (Q_hyp_) and absolute resting flow (Q_rest_), using a cut-off of 2.5 for pathological values (21). Microvascular Resistance Reserve (MRR) was calculated with the previous validated formula (17):


$$\begin{array}{l} MRR=CFR\;\cdot \frac{P_{a-rest}}{P_{d-hyp}} \end{array}$$

### Echocardiography

Patients underwent transthoracic echocardiography (TTE), as part of their routine clinical care, performed using a high-quality ultrasound machine (GE E95 or GE S70, GE Healthcare Horten, Norway) with a 3.5-MHz-phased array transducer (M5S). All images were stored for offline analysis by a cardiologist experienced in cardiac imaging, blinded to the invasively obtained data. Data were analyzed offline using dedicated software (EchoPAC PC SW-Only, version 202, GE Healthcare, Milwaukee, WI, USA). All patients had a comprehensive 2D echocardiographic assessment according to the European Association of Cardiovascular Imaging recommendations (22). Left ventricular ejection fraction (LVEF) was calculated using Simpson's biplane method. LV diastolic function was assessed by E, e' velocities, E/e', left atrial volume index (LAVi), and tricuspid regurgitation velocity. Determination of LV diastolic function was made using the algorithm proposed by the guidelines (23). Left atrial reservoir strain (LASr) was defined as the first peak positive deflection and represented the LA reservoir function. The LASr was calculated as the mean longitudinal strain in two apical views (4 and 2 chambers) using R-R gating as the zero-reference point.

### Statistics

Data distribution was assessed visually with histograms or with Shapiro-Wilk test as appropriate.

Baseline characteristics are presented as numbers (%) for categorical variables and as means ± standard deviation for continuous variables. Differences between groups were analyzed using the *t*-test or the Mann–Whitney *U*-test for continuous variables, and the chi-square test or the Fisher's exact test for categorical variables, as appropriate. Hemodynamic indices at rest and during hyperemia are presented as median and interquartile range [IQR] and compared with the Mann–Whitney *U*-test. For a comparison of more than two groups means one-way ANOVA or Kruskal-Wallis test were used as appropriate. Analyses were performed with R version 3.5.2 (R Foundation for Statistical Computing, Vienna, Austria). *P* < 0.05 was considered statistically significant.

## Results

### Baseline and Clinical Characteristics

Out of 188 patients screened for inclusion, continuous intracoronary thermodilution of the LAD was performed in 138 patients with suspected ANOCA. Fifteen patients were excluded because the clinical presentation was an acute coronary syndrome (*n* = 11) or because of a history of previous MI in the LAD (*n* = 4). Fifteen more patients were excluded because of obstructive coronary artery disease (DS>50%). The final population consisted of 108 patients, 21 in DM cohort and 87 in the control group. Mean age was 65.5 ± 11.05 and 62.8 ±11.0 years in the DM cohort and in non-diabetic patients respectively (*p* = 0.33). Eighty-six patients (79.6%) were male (16 [72.6%] in the DM cohort and 70 [80.5%] in the control group; *p* = 0.763). Among the 21 patients with diabetes mellitus, type I diabetes mellitus was detected in 3 patients. The median diabetes duration was 8.5 (3–10) years. Baseline and clinical characteristics were similar among the two groups and are shown in Table 1.

**Table 1:** Patients characteristics.

	**Non Diabetic (*N* = 87)**	**Diabetic (*N* = 21)**	**Total (*N* = 108)**	***p***
Age	62.85 ± 10.99	65.48 ± 11.05	63.36 ± 11.00	0.332
Male Sex	70 (80.5)	16 (76.2)	86 (79.6)	0.763
BMI	27.27 ± 3.54	30.90 ± 5.76	27.98 ± 4.28	0.009
Smoking	34 (39.1)	9 (42.9)	43 (39.8)	0.806
Hypertension	46 (52.9)	14 (66.7)	60 (55.6)	0.330
Dyslipidemia	68 (78.2)	19 (90.5)	87 (80.6)	0.355
Angina Class				0.271
CCS 0	21(24.4)	2 (9.5)	23 (21.5)	
CCS 1	47 (54.7)	13 (61.9)	60 (56.1)	
CCS 2	17 (19.8)	5 (23.8)	22 (20.6)	
CCS 3	1 (1.2)	1 (4.8)	2 (1.9)	
GFR				0.076
**>** 90 mL/min	10 (11.5)	2 (9.5)	12 (11.1)	
60–90 mL/min	71 (81.6)	14 (66.7)	85 (78.7)	
<60 mL/min	6 (6.9)	5 (23.8)	11 (10.2)	
Previous PCI	24 (27.6)	10 (47.6)	34 (31.5)	0.114
Previous MI	11 (12.6)	5 (23.8)	16 (14.8)	0.301
Aspirine	45 (51.7)	12 (57.1)	57 (52.8)	0.808
Anti P_2_Y_12_	17 (19.5)	1 (4.8)	18 (16.7)	0.188
Anticoagulation	6 (6.9)	4 (19.0)	10 (9.3)	0.101
ACEI/ARB	31 (35.6)	12 (57.1)	43 (39.8)	0.085
CCB	15 (17.2)	3 (14.3)	18 (16.7)	1.000
Statin	61 (70.1)	18 (85.7)	79 (73.1)	0.179
BB	27 (31.0)	4 (19.0)	31 (28.7)	0.420
Nitrates	3 (4.4)	3 (20.0)	6 (7.2)	0.069
Antiang	1 (1.5)	1 (6.7)	2 (2.4)	0.331
Metformin	0 (0)	17 (81)	17 (81)	<0.001
Sulfonylureas	0 (0)	6 (28.6)	6 (28.6)	<0.001
DPP-4 Inhibitors	0 (0)	1 (4.8)	1 (4.8)	<0.001
GLP-1 Agonist	0 (0)	1 (4.8)	1 (4.8)	<0.001
Insulin	0 (0)	8 (39.1)	8 (39.1)	<0.001
Glucose	NA	144.05 ± 51.25	144.05 ± 51.25	<0.001
HbA1c (%)	NA	6.73 (0.86)	6.73 (0.86)	NA
HbA1c (mmol/L)	NA	50.00 (9.40)	50.00 (9.40)	NA

*Continuous variables are presented as mean ± SD; categorical variables as number (%)*.

*BMI, Body Mass Index; CCS, Canadian Cardiovascular Society Angina Class; GFR, Glomerular Filtration Rate; PCI, Percuaneous Coronary Intervention; MI, Myocardial Infarction; ACEI, Angiotensin-converting enzyme; Angiotensin II Receptor Blockers; DPP-4, Dipeptidyl Peptidase 4; GLP, Glucagon-like peptide-1*.

### Hemodynamics

Angiographically, the DS% among the two groups were similar (27.48 ± 17.40 % in the DM group vs. 21.29 ± 14.36 % in the control group, *p* = 0.130). The median FFR value was 0.83 [0.79–0.87] without any significant difference between the two groups (0.82 [0.80–0.86] in the DM group vs. 0.84 [0.79–0.88] in the control group; *p* = 0.380). Absolute resting and hyperemic flow in the LAD were similar between diabetic and non-diabetic patients (resting flow: 78.62 [67.29–99.60] ml/min in the DM cohort vs. 71.62 [56.42–88.71] ml/min in the control group, *p* = 0.254; hyperemic flow: 199.28 [141.02–243.77] and 199.24 [162.69–264.44] for diabetic and non-diabetic patients respectively, *p* = 0.330). Similarly, resting and hyperemic resistances did not change significantly between the two groups (resting R: 977.53 [848.74–1,233.41] WU in the DM cohort vs. 1,135.14 [894.96–1,409.12] WU in the control group, *p* = 0.304; hyperemic R: 199.28 [141.02–243.77] vs. 199.24 [162.69–264.44] for diabetic and non-diabetic patients respectively, *p* = 0.507). Furthermore, both flow and resistance normalized for FFR were not significantly different between groups (Table 2).

**Table 2:** Angiographic and hemodynamic characteristics.

	**Non Diabetic (*N* = 87)**	**Diabetic (*N* = 21)**	**Total (*N* = 108)**	***p***
DS (%)	21.29 ± 14.36	27.48 ± 17.40	22.49 ± 15.11	0.130
FFR	0.84 [0.79–0.88]	0.82 [0.80–0.86]	0.83 [0.79 – 0.87]	0.380
Q_rest_ (mL/min)	71.62 [56.42–88.71]	78.62 [67.29–99.60]	71.87 [56.44, 89.56]	0.254
Q_rest−N_ (mL/min)	77.58 [63.11, 96.96]	85.64 [72.97, 107.26]	79.00 [63.86, 100.66]	0.255
R_μ−*rest*_ (WU)	1135.14 [894.96, 1409.12]	977.53 [848.74, 1233.42]	1107.99 [890.79, 1407.79]	0.304
Q_hyp_ (mL/min)	199.24 [162.69, 264.44]	199.28 [141.02, 243.77]	199.26 [156.47, 255.37]	0.350
Q_hyp−N_ (mL/min)	249.53 [199.07, 310.19]	229.90 [164.92, 285.47]	247.56 [196.97, 303.35]	0.478
R_μ−*hyp*_ (WU)	361.93 [299.51, 436.06]	390.07 [327.10, 450.22]	371.36 [301.57, 444.33]	0.507
CFR	2.88 ± 0.82	2.38 ± 0.61	2.78 ± 0.81	0.006
MRR	3.48 ± 1.02	2.79 ± 0.87	3.34 ± 1.03	0.004
CFR <2.5	30 (34.5%)	14 (66.7%)	44 (40.7%)	0.012
R_epi_ (WU)	73.24 [46.52, 103.23]	82.04 [63.64, 113.85]	74.10 [47.75, 105.96]	0.176
R_tot_ (WU)	434.20 [354.33, 536.08]	466.69 [412.94, 562.77]	456.89 [365.97, 537.31]	0.236

*Continuous variables are presented as mean ± SD or median [IQR]*.

*DS, Diameter Stenosis; FFR, Fractional Flow Reserve; Q_rest_, Resting Flow; Q_rest−N_, Normalized Resting Flow (Q_rest_/FFR); R_μ−rest_, Absolute Microvascular Resistance at Rest; Q_hyp_, Hyperemic Flow; Q_hyp−N_, Normalized Hyperemic Flow; R_μ−hyp_, Absolute Microvascular Resistance; CFR, Coronary Flow Reserve; MRR, Microvascular resistance Reserve; R_epi_, Epicardial Resistance (= P_a_ − P_d_)/Q); R_tot_, Total Coronary Resistance (= P_a_/Q)*.

**Figure 2:**
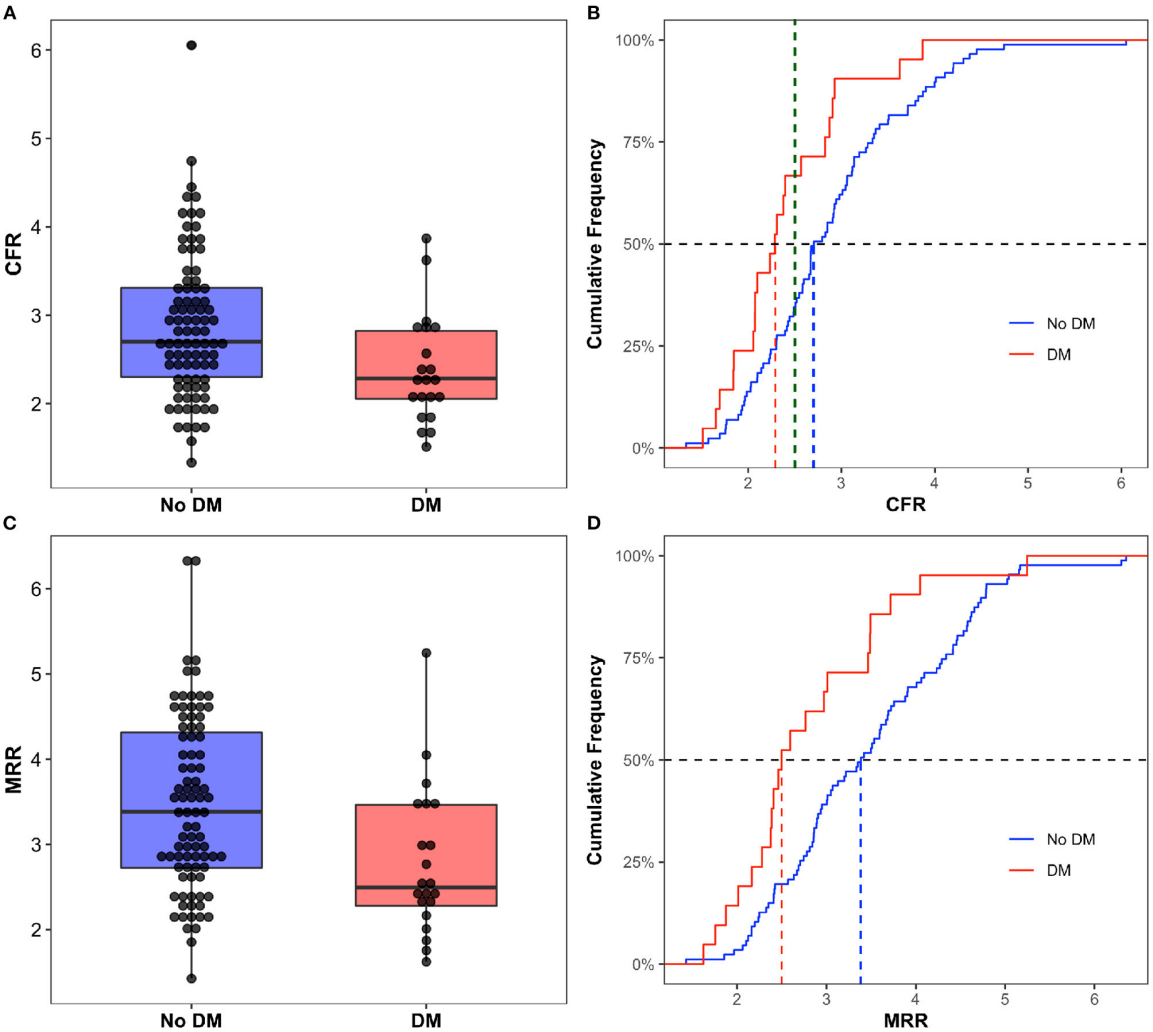
Box Plot and Cumulative Frequency of CFR **(A,B)** and MRR **(C,D)** in diabetic and non-diabetic patients. In the **(B)** the dashed green line corresponds to a CFR = 2.5. DM, Diabetes Mellitus; CFR, Coronary Flow Reserve; MRR, Microvascular resistance Reserve.

**Figure 3:**
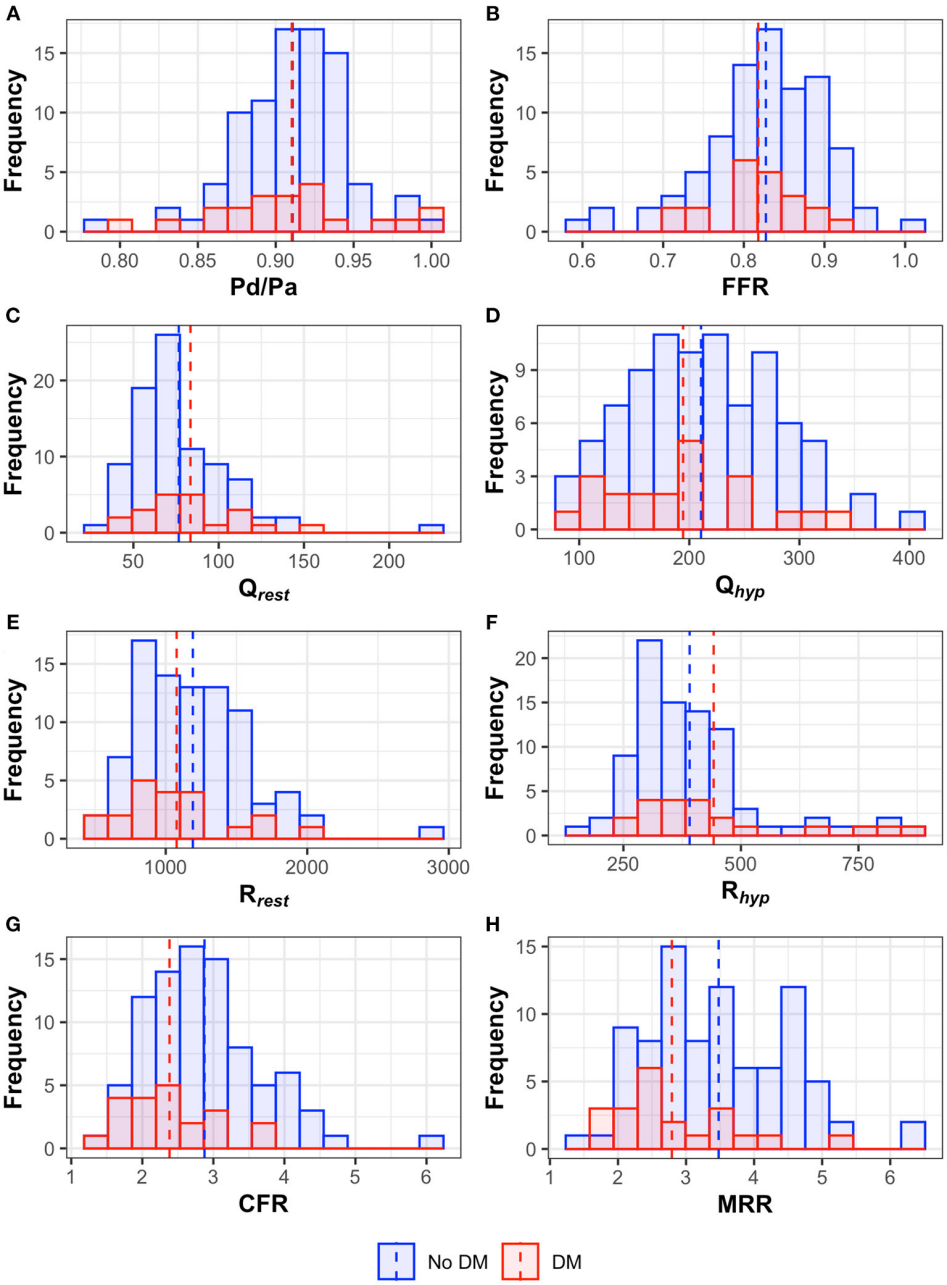
Histograms and median values (dotted line) of the hemodynamic parameters [**(A)** Pd/Pa, **(B)** fractional flow reserve – FFR, **(C)** resting flow, **(D)** hyperemic flow, **(E)** resting resistance, **(F)** hyperemic resistance, **(G)** coronary flow reserve – CFR, **(H)** microvascular resistance reserve – MRR] in both diabetic and non-diabetic patients.

Interestingly the CFR was significantly lower in the DM cohort (2.37 ± 0.60 vs. 2.88 ± 0.82 in the DM and non-DM cohort respectively, *p* < 0.05) as well as the MRR (2.79 ± 0.87 in the DM group vs. 3.48 ± 1.02 in the control group, *p* < 0.05) (Figure 2). In the overall population, 40.7% of the patients had a CFR <2.5 and the proportion was significantly higher in the DM group (14[66.7%] vs. 30[34.5%] in the non-diabetic group, *p* < 0.05). Angiographic and hemodynamic characteristics are summarized in Table 2. Distribution of the main hemodynamic parameters per groups is shown in Figure 3.

### Echocardiographic Characteristics

Echocardiographic data were available for 92 out of 108 patients. Sixteen cases were excluded from the original cohort for either poor acoustic window, sub-optimal EKG, or image quality for speckle-tracking analyses. The median time between TTE and index CAG was 19 [IQR 10–65] for non-diabetic and 27 [IQR 7–60] days for the diabetic cohort, respectively. Table 3 summarizes the echocardiographic characteristics of both groups. No significant differences were observed in most morphological and functional echocardiographic characteristics between the two groups. However, diabetic patients had a significantly greater E wave and average E/e' compared with non-diabetic group (both *p* < 0.05). Interestingly, diabetic patients had a significantly lower reservoir, contractile and conductive LAS (all *p* < 0.05).

**Table 3:** Echocardiographic characteristics.

	**Non Diabetic (*N* = 74/87)**	**Diabetic (*N* = 18/21)**	**Total (*N* = 92/108)**	***p***
BSA, (m^2^)	2 [1.85–2.12]	1.9 [1.85–2.3]	2 [1.85–2.16]	0.43
LVEDDi, (mm)	23.7 [22.2–25.8]	23.5 [21.6–25.6]	23.7 [22–25.8]	0.65
LVEDVi, (mm)	43 [36–55]	42 [34–48]	42.3 [39.4−53.9]	0.44
IVS, (mm)	11 [9–12]	11 [9–12]	10 [9–12]	0.47
PWT, (mm)	9 [8–10]	9 [8–10]	8 [9–10]	0.71
RWT	0.38 [0.33–0.42]	0.38 [0.33–0.43]	0.38 [0.33–0.42]	0.88
LVMi	85 [70–93]	75 [69–96]	80.8 [69.9–93.3]	0.67
2D BP LVEF, (%)	55 [55–60]	55 [54–59]	55 [55–60]	0.25
TAPSE, (mm)	23 (20-24)	22 [18–23]	23 [20–24]	0.14
E wave, (m/sec)	0.64 [0.5–0.8]	0.79 [0.64–1.01]	0.68 [0.52–0.81]	0.014
E/A, (ratio)	0.87 [0.7–1.2]	0.9 [0.8–1.2]	0.88 [0.7–1.2]	0.84
e' septal, (m/sec)	0.07 [0.05–0.08]	0.06 [0.05–0.08]	0.07 [0.05–0.08]	0.64
e', lateral, (m/sec)	0.09 [0.07–0.11]	0.09 [0.08–0.10]	0.09 [0.07–0.11]	0.76
e', average, (m/sec)	0.08 [0.06–0.10]	0.07 [0.06–0.09]	0.08 [0.06–0.1]	0.94
E/e' average	8.2 [6.6–10.3]	9.8 [8.7–12]	8.7 [7–11]	0.026
TR V max, (m/sec)	2.5 [2.4–2.7]	2.6 [2.5–2.7]	2.5 [2.4–2.7]	0.40
TR gradient, (mmHg)	30 [25–35]	30 [28–38]	30 [25–35]	0.24
LAVi, (ml/m^2^)	25.5 [20.2–31.6]	24.6 [21.2–30.2]	25.5 [21.2–31.2]	0.96
E/e' > 14	9 (12.3)	2 (11.1)	11 (12.1)	0.89
e' sep. <0.07 or e' lat. <0.1 m/s	47 (64.4)	12 (66.7)	59 (64.8)	0.86
LAVi > 34 ml/m2	14 (19.2)	4 (22.2)	18 (19.8)	0.77
TR vel. > 2.8	10 (13.7)	2 (11.1)	12 (13.2)	0.77
Diastolic function				0.50
Normal	55 (75.3)	14 (77.8)	69 (75.8)	
Indeterminate	12 (16.4)	3 (16.7)	15 (16.5)	
Dysfunction	6 (8.2)	2 (11.1)	8 (7.7)	
LASr, (%)	28 [24–35]	23 [17–26]	26 [22–34]	0.001
LASct, (%)	15 [12–18]	10 [7–13]	14 [11–17]	0.002
LAScd, (%)	13 [11–19]	11 [7–12]	12 [9–18]	0.004

*Continuous variables are presented as median (IQR); categorical ones as n (%)*.

*LVEDD, left ventricular end-diastolic diameter indexed to BSA; LVEDVi, left ventricular end-diastolic volume indexed to BSA; IVS, interventricular septum; PWT, posterior wall thickness; LVMi, left ventricular mass indexed to BSA; 2D LVEF, two-dimensional left ventricular ejection fraction; TAPSE, tricuspid annular plane systolic excursion; TR, tricuspid regurgitation; LAVi, left atrium volume index. LASr, left atrial reservoir strain; LASct, left atrial contraction strain; LAScd, left atrial conduit strain*.

## Discussion

The present study is the first to compare absolute coronary flow and microvascular resistance in diabetic vs. non-diabetic patients. The main findings of our study are the following: (1) patients with DM have a lower CFR as compared to non-diabetic patients and the proportion of patients with an abnormal CFR is higher among diabetic patients; (2) the MRR is significantly lower in diabetic patients; (3) diabetic patients presented decreased LASr compared to the non-diabetic patients.

In the heart, microcirculatory dysfunction usually precedes structural myocardial changes; thus the evolving ability to an early assessment of CMD holds great potential for risk stratification and patient therapy (24). Traditionally, both intracoronary bolus thermodilution (measuring the flow as mean transit time—Tmn, in sec.) and intracoronary doppler (measuring flow velocities in cm/s) provide surrogate indexes of flow. Yet, these latter present some technical challenges and bear significant patient and operator-related variability hampering their widespread clinical adoption (12, 25–27). In our study, we used continuous intracoronary thermodilution, which is operator-independent, and it allows a direct volumetric quantification of the blood flow (in mL/min) (28).

It must be considered that, since this is a retrospective study, diabetic patients were not compared with a cohort of healthy control subjects but rather to a population of patients with a history of angina or ischemic heart disease (IHD); yet in the non-diabetic cohort 54.7% of patients had class 1 angina, 27.6% had a previous PCI and 12.6% had a previous MI. Nevertheless, the burden of epicardial atherosclerosis was similar among the two groups as demonstrated by the DS% and the FFR.

### Microvascular Dysfunction in Diabetic Patients

The relatively low values of FFR in the overall population as compared to the DS% may be attributed to either the diffuseness of the epicardial atherosclerosis, the distal position of the pressure wire and the presence of the RayFlow in the vessel, as previously demonstrated (15). Moreover, R_epi_ and FFR values, did not significantly differ between groups, confirming that epicardial conductance were comparable between the two populations.

In the absence of obstructive epicardial disease, the lower CFR values measured in the diabetic cohort are likely to be related to presence of microvascular dysfunction. However, when epicardial arteries are not completely normal—as in our cohort -, CFR may not allow a precise detection of microvascular disease (29, 30); thus for a more accurate evaluation of microvascular compartment we adopted MRR, which is a novel index, specific for microvasculature, independent of epicardial stenosis and myocardial mass (17). MRR was significantly lower in the diabetic cohort thus reflecting an impaired vasoreactivity of the microcirculation in these patients.

### Mechanisms of Microvascular Dysfunction

Microvascular dysfunction, in the absence of obstructive epicardial disease, can be driven by either an increased basal flow (with decreased R_rest_), a decreased hyperemic flow (with increased R_hyp_) or a combination of both (31–33). The primary mechanism leading to the CFR impairment in diabetic patients is still unclear; Picchi et al., by using bolus thermodilution, showed that a decreased CFR in diabetic patients was primarily related to a decrease in rest Tmn, thus reflecting an increased resting flow (11); similarly, PET-based study demonstrated a decrease in CFR in diabetic patients due to an increase of resting myocardial blood flow (34). Conversely, other studies based on PET (35, 36) or invasive Doppler velocity measurements (37), found that the CFR in these patients was decreased due to a decrease in hyperemic flow. Interestingly, Sezer et al. (38) by using transthoracic Doppler echocardiography, found a bimodal pattern of microvascular impairment in diabetic patients consisting in increased resting blood flow in the early stage of the disease and decreased hyperemic response in long-standing (>10 years) diabetes. In our study, absolute flow and microvascular resistance at rest and during hyperemia did not differ significantly between patients with and without diabetes but, the lower MRR in the diabetic cohort is likely the result of a combination of both an increased resting flow and a decreased hyperemic flow (Figure 3).

### Microvascular Dysfunction and Left Atrial Strain

Regardless of the mechanisms underlying the microcirculatory impairment, these changes, as in other organs, precede structural changes. As outlined by Paulus et al., diabetes and obesity by inducing a pro-inflammatory state, lead to a microvascular dysfunction that, ultimately, by inducing LV remodeling determine a diastolic dysfunction (39). LASr, has shown to be an early predictor of LV diastolic dysfunction and a reduced LASr has been correlated with CMD (40, 41). Interestingly, in our study, diastolic function was mainly preserved in both diabetic and non-diabetic patients with a LASr that was lower in diabetic patients; this finding is probably linked to the worse microvascular function in this subpopulation which might precede the onset of overt diastolic dysfunction.

### Limitations

Our results should be interpreted considering some limitations. First, this was a single-center retrospective study with a relatively small number of patients, thus our findings will need to be confirmed in further prospective investigations with a larger sample size. Second, we investigated the coronary physiology only in the LAD thus the results cannot be generalized to other coronary territories. Third, the lack of data concerning plasma glucose in patients without diabetes does not allow to extend our results to patients with pre-diabetes. Lastly, the patients included in the present study were heterogeneous in terms of their diabetic status and stage of glucose metabolic impairment.

## Conclusion

In our study, CFR and MRR were significantly impaired in patients with diabetes mellitus. Furthermore, the lower LASr in the diabetic cohort may reflect a subclinical diastolic dysfunction subsequent to the CMD. MRR, derived by continuous intracoronary thermodilution, provides a reliable and operator-independent assessment of coronary and microvasculature and is independent from epicardial stenosis and myocardial mass; this novel index might potentially facilitate widespread clinical adoption of invasive physiologic assessment of suspected microvascular disease.

## Data Availability Statement

The raw data supporting the conclusions of this article will be made available by the authors, without undue reservation.

## Ethics Statement

The studies involving human participants were reviewed and approved by Ethics Committee OLV Aalst. The patients/participants provided their written informed consent to participate in this study.

## Author Contributions

EG carried out the statistical analysis. EG and PP wrote the first draft of the manuscript. AC, KB, DF, GE, DB, EF, DM, and NM collated data. EB, BD, CC, MP, JB, MV, EW, and JS corrected and approved the revisions and final version of the manuscript. EG, PP, EB, and BD are responsible for the conception, funding, and design of the study. EB is the guarantor of this work and, as such, had full access to all the data in the study and takes responsibility for the integrity of the data and the accuracy of the data analysis. All authors contributed to the article and approved the submitted version.

## Funding

PP, GE, DF, and JS are supported by a research grant from the CardioPaTh PhD Program. The funder was not involved in the study design, collection, analysis, interpretation of data, the writing of this article or the decision to submit it for publication.

## Conflict of Interest

CC reports receiving research grants from Biosensor, Coroventis Research, GE Healthcare, Medis Medical Imaging, Pie Medical Imaging, Cathworks, Boston Scientific, Siemens, HeartFlow Inc. and Abbott Vascular; and consultancy fees from Heart Flow Inc, Opsens, Pie Medical Imaging, Abbott Vascular and Philips Volcano. BD has a consulting relationship with Boston Scientific, Abbott Vascular, CathWorks, Siemens, and Coroventis Research; receives research grants from Abbott Vascular, Coroventis Research, Cathworks, Boston Scientific; and holds minor equities in Philips-Volcano, Siemens, GE Healthcare, Edwards Life Sciences, HeartFlow, Opsens, and Celiad. EB declares speaker's fees from Abbott Vascular, Boston Scientific and GE. The remaining authors declare that the research was conducted in the absence of any commercial or financial relationships that could be construed as a potential conflict of interest.

## Publisher's Note

All claims expressed in this article are solely those of the authors and do not necessarily represent those of their affiliated organizations, or those of the publisher, the editors and the reviewers. Any product that may be evaluated in this article, or claim that may be made by its manufacturer, is not guaranteed or endorsed by the publisher.
